# A Preliminary Study on Identifying the Predator Community of Invasive *Bactericera cockerelli* (Hemiptera: Triozidae) and Developing Molecular Identification Tools for Testing Field Predation

**DOI:** 10.3390/insects16020179

**Published:** 2025-02-07

**Authors:** Shovon Chandra Sarkar, Stephen Paul Milroy, Wei Xu

**Affiliations:** Food Futures Institute, Murdoch University, Murdoch, WA 6150, Australia

**Keywords:** invasive pest, generalist predators, predation, molecular detection, predator–prey relationship

## Abstract

The tomato potato psyllid *Bactericera cockerelli* (Hemiptera: Triozidae) is a significant insect pest of Solanaceae. In early 2017, it was first detected in Perth, Western Australia. The objective of this work was to identify predator species of *B. cockerelli* occurring in fields of Solanaceae in Western Australia. Predatory insects and arachnids were sampled using sweep netting in some of the major Solanaceae-growing regions in the south-west of Western Australia in 2021 and 2022. Several laboratory feeding trials were conducted to develop PCR primers that could detect the DNA of *B. cockerelli* in predators that had fed on *B. cockerelli* rather than on alternative diets. The primers were then used to screen predators collected from the field to identify those that had been feeding on *B. cockerelli*. In the two years of field sampling, the predators collected represented a broad taxonomic range. The most abundant predator was green lacewing followed by ladybirds. Further, we analysed predators belonging to seven insect taxa (one Neuroptera, two Hemiptera and four Coleoptera) for the presence of *B. cockerelli* DNA. We found that 45% of the individual insects from all taxa that we caught were positive for *B. cockerelli* DNA, and Coleopteran predators showed the highest rate of positive results. This is the first report confirming predation on invasive *B. cockerelli* by the resident predator community in the field in Australia.

## Simple Summary

This study investigated generalist predators in Solanaceous crop fields across Western Australia to understand their role in controlling the invasive pest *Bactericera cockerelli*. A diverse range of predator species, including insects from Neuroptera, Coleoptera, Diptera and Hemiptera, as well as spiders, was identified. Laboratory feeding trials and molecular analysis confirmed that many of these predators consumed *B. cockerelli* in the field. Notably, green lacewings and ladybirds were the most abundant predators, with capsicum fields supporting the largest populations due to floral resources. Molecular techniques revealed that 45% of the tested predators consumed the pest, with Coleopteran predators showing the highest positivity rates, followed by Neuroptera and Hemiptera. Predatory spiders were also common, though their populations varied between years. This study emphasizes the utility of molecular tools for monitoring predation and suggests that these predators could play a key role in integrated pest management strategies for *B. cockerelli* in Australia.

## 1. Introduction

The tomato potato psyllid, *Bactericera cockerelli* (Šulc) (Hemiptera: Triozidae), is one of the biggest threats for solanaceous crops [1,2,3,4]. Native to southern North America [5], it has recently become an international concern. In 2017, *B. cockerelli* was first reported in Western Australia as an invasive pest species [6]. The overwintering ability of *B. cockerelli*, the favourable climatic conditions and the presence of numerous cultivated and wild solanaceous hosts facilitated their establishment around the city of Perth. The tomato potato psyllid can damage host plants through phloem feeding as well as by acting as a vector of the alphaproteobacterium *Candidatus* Liberibacter solanacearum (CLso), which can cause “zebra chip” disease in potato [7,8,9]. To date, there has been no record of zebra chip disease in mainland Australia [6]. However, the established population of the CLso vector, *B. cockerelli,* in Western Australia raises concern about the potential spread of zebra chip disease if CLso is introduced.

*Bactericera cockerelli* is a polyphagous pest, with short developmental times, high reproductive potential and flying capability [2,3,10,11], which make it difficult to manage in cropping systems. To control *B. cockerelli* in the field, growers rely on the repeated use of chemical insecticides [9,12,13,14]. Repeated application of insecticides may kill non-target species including natural enemies [9,14]. It may also increase the risk of residue problems, environmental contamination, species displacement and disruption of IPM systems [15,16]. Moreover, resistance to insecticides (e.g., neonicotinoid, abamectin, endosulfan) has already been recorded in *B. cockerelli* populations in the USA and Mexico [17,18,19,20,21], raising concerns about the overuse of other chemicals. Therefore, the possibility of using natural enemies for augmentative biological control is gaining popularity [22,23,24].

Invasive pest species, such as *B. cockerelli*, often possess high reproductive capacities, competitive advantages for resources and the ability to escape predation from their natural enemies when invading new regions [25]. To develop a sustainable invasive pest management program, understanding the natural enemy community in the invaded regions becomes crucial [26]. Generalist predators are known for their capacity to regulate herbivorous arthropod populations in various agroecosystems and can use an invasive pest as an alternative food source [27,28,29]. Although parasitoids may impact an invasive pest, this is usually after their establishment, mostly when the invasive species is closely related to their normal host species [30,31]. In such scenarios, resident generalist predators can play a vital role in preventing the invasion of new crop areas by alien pests and reducing the overall pest populations within the fields [32,33]. In our recent review, a diversity of species was identified that are known to be predators of *B. cockerelli* in other countries and were already resident in Australia [34].

Molecular techniques are commonly employed in insect ecology to detect DNA traces of prey species in predators [35,36,37,38,39,40]. DNA analysis has proven useful in identifying predators for the development of augmentative biological control strategies against significant agricultural pests [41,42,43], characterizing generalist predator food webs in agroecosystems [44,45] and assessing the impacts of exotic predators on native competitors or prey species [26,46].

Polymerase chain reaction (PCR) is widely used in analysing predators to identify their prey [27,47,48]. It allows the rapid and specific identification of the DNA of the target prey species [32]. Importantly, this method can only detect undigested DNA when predators are actively feeding on the pest species. This method also helps to identify novel predator species of a pest. For example, Peterson et al. [49] confirmed *Orius insidiosus* Say (Hemiptera: Anthocoridae) as a predator of the corn pest *Helicoverpa zea* Boddie (Lepidoptera: Noctuidae) using molecular gut content analysis.

In this research, we employed PCR to identify predator species actively feeding on *B. cockerelli* in solanaceous crops in Western Australia. For speed of processing, we used the whole corpus of the predator for analysis. DNA primers specific for *B. cockerelli* were developed and tested through laboratory feeding trials. The primers’ specificity was assessed by testing them against closely related psyllid species and other local prey as well as co-occurring predator species. Moreover, to ensure that we were detecting predators that were actively feeding on *B. cockerelli*, we determined the time after feeding that it took for the DNA of the prey to become non-detectable in the predator corpus.

The objective of the present study was to identify key predators of the invasive pest *B. cockerelli* in the Western Australian agroecosystem. Field sampling was conducted first to identify the potential predators of *B. cockerelli* on Solanaceae fields. These predators were then analysed to investigate their active predation on *B. cockerelli*.

## 2. Material and Methods

### 2.1. Field Sampling of Predators and Identification

Samplings were conducted in 2021 and 2022 on well-established crops like capsicum (*Solanum annuum* L.) and potato (*Solanum tuberosum* L.) as well as on the weed black-berry nightshade (*Solanum nigrum* L.) present in the fields. Sampling was conducted at one site in 2021 and at five sites in 2022 in commercial fields from Bullsbrook (31.67° S, 115.99° E, 40 km north of Perth) to Yallingup (33.67° S, 115.03° E, 200 km south of Perth), Western Australia (WA) (Table 1). The aim was to cover a wide crop area to identify potential predators of *B. cockerelli* occurring in WA agroecosystems. All samplings were conducted at maximum vegetative growth at a time when high numbers of *B. cockerelli* were expected.

The predators were sampled using two methods: (i) sweep netting of crops; (ii) visual surveys on nightshade (in situ collection for 1 h between 10.00 a.m. and 2.00 p.m. from randomly selected plants). At each site, the samples from weeds were combined with the samples from the crop. Sweep netting with a 38 cm diameter net was used to sample insects in the crop foliage along the edge of the fields. Each field was sampled along 15 transects, with each transect spanning 15–20 m. Each meter along the transect, the sweep net was swept through an arc of 180°. Fifteen samples were obtained from each site on each sampling date over the sampling period. For the nightshade, visual count and collection were conducted on 15–25 plants per sampling sites. The samples were immediately placed into labelled plastic jars containing 96% ethanol. They were then brought to the laboratory at Murdoch University in Perth and stored at −80 °C for the sorting, identification and counting of predatory insects.

The identification of the field-collected predators, those that are known to attack a range of various pest species and likely to prey on *B. cockerelli*, was based on morphological characteristics (body shape, coloration, mouthparts, wings, leg structure and abdominal characteristics) observed under a stereo microscope [12]. Once identified, each individual predatory insect was preserved in a 1.5 mL Eppendorf microtube containing 96% ethanol and stored at −80 °C to maintain DNA integrity and prevent contamination [50]. These preserved specimens were subsequently used for molecular analysis to detect the presence of the DNA of *B. cockerelli*.

### 2.2. Development of a PCR Assay for Predators of B. cockerelli

A PCR method was developed to identify individual predators that had recently consumed *B. cockerelli*, enabling the identification of the predator species involved and the prevalence of predation. Firstly, species-specific PCR primers for the identification of *B. cockerelli* were designed based on cytochrome c oxidase subunit 1 (CO1) sequence differences relative to other common prey and predator species present in the fields where *B. cockerelli* was found. The prey species considered were *Myzus persicae* Sulzer (Hemiptera: Aphididae), *Macrosiphum euphorbiae* Thomas (Hemiptera: Aphididae), and *Brevicoryne brassicae* L. (Hemiptera: Aphididae); the predator species considered were *Coccinella transversalis* Fabricius (Coleoptera: Coccinellidae), *Hippodamia variegata* Goeze (Coleoptera: Coccinellidae), and *Mallada signatus* Schneider (Neuroptera: Chrysopidae). The sequences of each insect used in primer design were acquired from the NCBI nucleotide database. The primers (forward 5′-TTTCAAAATGTTAGTAAG-3′; reverse 5′-ATACGAAGATGTATATGT-3′) were designed using the Clustal Omega’s multiple sequence alignment feature, based on sequences available in GenBank, accession number KU501214) [51].

**Figure 1:**
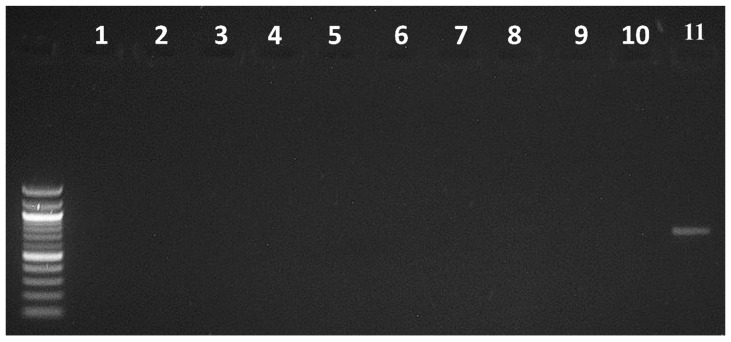
Specificity of PCR detection assays for *B. cockerelli* DNA (11) when tested against other psyllid species (1–10). Here, 1: *A. credoensis*; 2: *C. bipartite*; 3: *G. brimblecombei*; 4: *A. bundoorensis*; 5: *B. occidentalis*; 6: *S. marginata*; 7: *B. tremblayi*; 8: *B. trigonica*; 9: *B. nigricornis*; 10: *A. dobsonii*; and 11: *B. cockerelli*.

**Figure 2:**
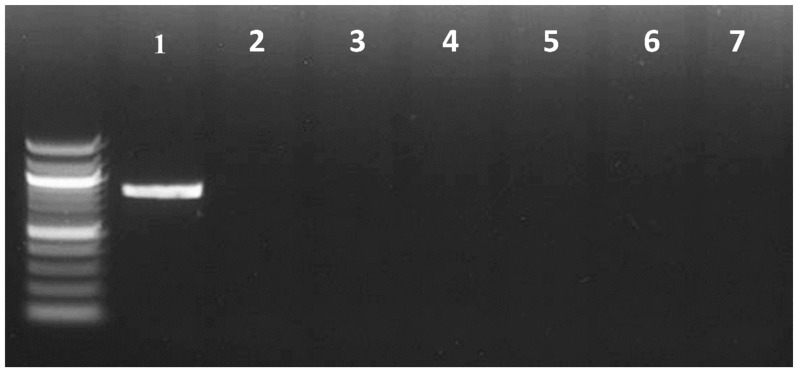
Specificity of PCR detection assays for *B. cockerelli* DNA (1) when tested against aphid species (2–4) and predator species (5–7). Here, 1: *B. cockerelli*; 2: *M. persicae*; 3: *M. euphorbiae*; 4: *B. brassicae*; 5: *C. transversalis* larvae feed on *B. brassicae*; 6: *H. variegata* larvae feed on *M. persicae*; and 7: *M. signatus* larvae fed on *M. persicae*.

To confirm the PCR method’s specificity and selectivity in distinguishing *B. cockerelli* from other insects, *B. cockerelli* DNA was used as a positive control, while the DNA of the other psyllid species (*Acizzia credoensis* Taylor & Kent (Hemiptera: Psyllidae), *Ctenarytaina bipartite* Burckhardt (Hemiptera: Psyllidae), *Glycaspis brimblecombei* Moore (Hemiptera: Psyllidae), *Anoeconeossa bundoorensis* Taylor & Burckhardt (Hemiptera: Aphalaridae), *Blastopsylla occidentalis* Taylor (Hemiptera: Psyllidae), *Schedotrioza marginata* Taylor (Hemiptera: Triozidae), *Bactericera tremblayi* Wagner (Hemiptera: Triozidae), *Bactericera trigonica* Hodkinson (Hemiptera: Triozidae), *Bactericera nigricornis* Förster (Hemiptera: Triozidae) and *Aacanthocnema dobsonii* Froggatt (Hemiptera: Triozidae)) (Figure 1) and insects that were used in primer design were used as negative controls (Figure 2). This allowed for the evaluation of PCR’s ability to accurately differentiate *B. cockerelli* from the selected insect species.

DNA extraction was performed using DNeasy Blood & Tissue Kits (QIAGEN, Valencia, CA, USA) according to product instructions. Whole insects including both prey and predator were used for DNA extraction. The insects were macerated in Buffer ATL using tissue grinders and incubated with proteinase K at 56 °C for 1–1.5 h. The final yield of DNA was quantified in µg/µL in each extraction using a NanoDrop Microvolume Spectrophotometer (Thermo Fisher Scientific, Waltham, MA, USA). All DNA samples were stored at −20 °C.

PCR amplification was performed using nuclease-free biotechnology-grade water (Fisher Biotech, Wembley, WA, Australia), 5X Phusion HF buffer, 10 mM dNTPs, Phusion DNA polymerase (New England Biolabs, Ipswich, MA, USA), 1.25 μL of each primer and 1 μL of insect DNA in a 25 μL reaction. PCR was performed in a Veriti Thermal Cycler (Thermo Fisher Scientific Inc., Waltham, MA, USA) or an Eppendorf^®^ Mastercycler (Sigma-Aldrich, St. Louis, MI, USA). The program cycle was 98 °C for 30 s followed by 35 cycles as follows: 98 °C for 10 s, 55 °C for 30 s, 72 °C for 30 s, and finally 72 °C for 2 min. Electrophoresis (90 V) was used to confirm amplification with 10 µL of 100 bp ladder (New England Biolabs) and PCR product (10 µL) in 1.0% ultrapure agarose (Thermo Fisher Scientific Inc., Waltham, MA, USA) stained with SYBR Safe DNA Gel Stain (0.1 mg/µL, Thermo Fisher Scientific Inc., Waltham, MA, USA). The gel was visualized and photographed using a Transilluminator (Fisher Biotech, Australia).

To validate the ability of the PCR method to differentiate between predators that consumed *B. cockerelli* and those that did not, feeding trials were conducted. During our field surveys, some predators were observed preying on *B. cockerelli* [34], and some were more abundant than others. On this basis, the adult stages of the two most abundant ladybird species and the larval stage (3rd instar) of the most abundant lacewing species were selected for the feeding trials. Ladybirds, *H. variegata* and *C. transversalis*, were collected from canola fields in Northam, Western Australia, Australia, while a lacewing *M. signatus* culture was purchased from BioResources Pty Ltd. (Queensland, Australia). These predators were fed on *Myzus persicae* Sulzer (Hemiptera: Aphididae) on tomato plants in a greenhouse. For each feeding unit, a water-saturated filter paper was placed at the bottom of a 5 cm diameter Petri dish, and a tomato leaf disk (4 cm diameter) was placed upside down on the filter paper. In the Petri dish, the predators were individually feed ad libitum (50–60 prey per Petri dish) on a single prey species (either *B. cockerelli* or *M. persicae*). To ensure all previous food had been digested, all predators were starved for 24 h before being introduced to the Petri dish. The predators were left in the dish with access to the prey species for 2 h and then immediately transferred into 1.5 mL Eppendorf microtubes containing 96% ethanol and stored at −20 °C. DNA was then extracted from the predators and tested using the PCR method developed using the *B. cockerelli*-specific primers. Predators that feed on *M. persicae* were used as the negative control.

Furthermore, the time-specific sensitivity of the approach for the detection of *B. cockerelli* DNA from the predator’s corpus was assessed based on the time difference between removal from access to *B. cockerelli* and sampling for DNA extraction. As described in the previous paragraph, individual predators were starved and then feed ad libitum on a single prey species (*B. cockerelli* or *M. persicae*) in a 5 cm diameter Petri dish. After 2 hrs of feeding, the predators were removed carefully and placed individually into another Petri dish where no prey were present. This moment was considered time 0. After 0 h, 0.5 h, 1 h, 2 h, 6 h, 12 h and 24 h, individual predators were randomly selected and placed into a 1.5 mL Eppendorf microtube containing 96% ethanol, then stored at −20 °C for DNA extraction using the *B. cockerelli*-specific primers.

### 2.3. Molecular Analysis of Field-Collected Predators

The insects collected from the field (Table 1) were analysed using the approach outlined above. For each potential predator species, one individual was selected randomly form each sample for molecular analysis. The intention was to take ten individuals per species, but for some species, this was not possible. The final species tested and the number of individuals used were *Nabis* sp. adult (n = 8), *O. schellenbergii* adult (n = 6), *M. signatus* larvae (n = 10), *C. sexmaculata* adult (n = 2), *H. conformis* adult (n = 5), *C. transversalis* adult (n = 8), and *H. variegata* (n = 10). *B. cockerelli* DNA was used as the positive control. A product band presenting the same size as the positive control indicated that the individual had recently consumed *B. cockerelli*, within the last 6 h. Because spiders are not readily available for commercial purposes and are challenging to rear artificially, in contrast to *Creontiades* sp., also known as plant bugs, they were excluded from the molecular analysis in the present study.

To ensure the quality of DNA in each field sample tested, the restriction enzyme *Eco*R1-HF^®^ (NEB) was employed to digest the whole extracted DNA samples from each test insect, following the manufacturer’s instructions. A 50 μL solution containing insect DNA, buffer, and the restriction enzyme was incubated at 37 °C for 1 h. The enzyme was subsequently inactivated by placing the solution in an ice bath for 15 min. The DNA samples, along with the 1 kb Plus DNA molecular ladder, were loaded onto a 0.8% agarose gel stained with a DNA-specific dye in 1X TAE (Tris–acetate–EDTA) buffer. The DNA was then separated in a mini sub-cell at 70 V for 1 h (BIO-RAD). The resulting DNA bands were visualized under a UV transilluminator to examine the presence of DNA fragments.

The percentage of predator sensitivity to *B. cockerelli* DNA was analysed using a χ^2^ goodness-of-fit test with an expected response of 50% for both positive and negative predator sensitivity detection. This analysis was conducted in SPSS (version 29.0, SPSS Inc., Chicago, IL, USA).

## 3. Results

### 3.1. Identification of Predators

Samples from the field were screened to identify generalist predators known to prey on different insect pest species and expected to attack *B. cockerelli.* Six different families of spider and seven of insect were represented (Table 1). Among the sampled arthropods, six different spider families were identified, with Oxyopidae being the most prevalent, accounting for 56% of the total sampled spiders. Eleven different species of potential insect predators (n = 1653) were identified. In both years, Chrysopidae, a family of green lacewings, was the most abundant family, accounting for 79% (n = 1313) of the total number of potential predators. Coleoptera was the second most abundant (6% (n = 101) of total predators) predatory arthropod order across the two years. Among the four species of coccinellids identified, the transverse ladybird beetle, *C. transversalis* (male and female adults), made up 51% of the identified beetles across the two years, followed by the variegated ladybird beetle, *H. variegata* (male and female adults) (32%). Four families of Hemipteran predators (Anthocoridae, Miridae, Nabidae and Rhyparochromidae) were also present. The mirid bug *Creontiades* sp. was very abundant in capsicum in 2021 but much less so in 2022. A native hoverfly species, *Melangyna viridiceps* Macquart (Diptera: Syrphidae), was also recorded in both years. No predators were found in the second (capsicum) and third site (eggplant) in 2021.

### 3.2. Laboratory Study: Predator Feeding Trials

The designed primers amplified a specific band from *B. cockerelli* DNA with the expected size (832 bp). The DNA band was purified and sequenced, showing that it was the target *B. cockerelli* DNA COI sequence. However, this band was not amplified from DNA samples of other psyllid species and local aphids, as well as predator species feeding on local aphid species (Figure 1 and Figure 2), suggesting that the designed primers were specific and selective for detecting the *B. cockerelli* DNA from predators.

Five feeding trials were conducted to test for the ability of the method to detect the presence of *B. cockerelli* DNA in predators that had fed on the psyllid. These covered the two Coleoptera species that were most common in both years (*C. transversalis* and *H. variegata*) in both the larval and the adult stages, and one species of Neuroptera. Only the larvae were tested for this order, as the adults are not predatory. The two coccinellids corresponded to those used in our earlier studies [52,53]. The predators were provided with *B. cockerelli* as a prey. Subsequently, the predators were separated from direct contact with the psyllid and sampled at different time intervals to conduct molecular analysis and evaluate the detection of *B. cockerelli* DNA within their bodies (Table 2). In all cases, *B. cockerelli* DNA was detectable for 1 h after feeding. For *C. transversalis*, *B. cockerelli* DNA was detectable for 2 h after feeding. For *H. variegata*, the psyllid DNA was detectable for 1 h after feeding. In the case of *M. signatus* larvae, the psyllid DNA remained detectable for 6 h after feeding.

### 3.3. Molecular Analysis of Field-Collected Predators

**Figure 3:**
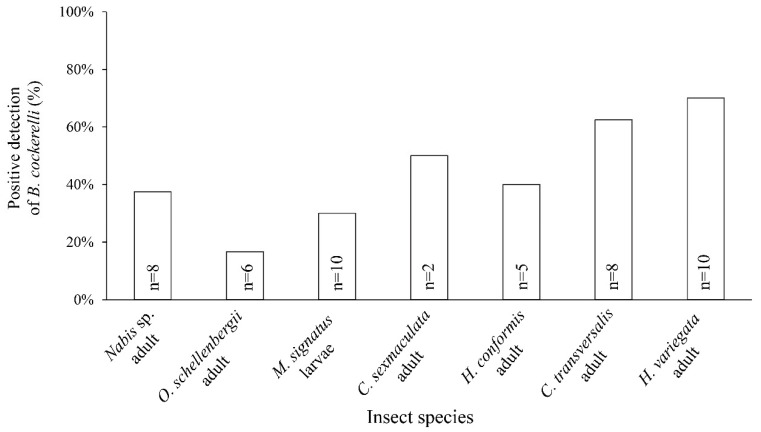
Percentage of samples that tested positive for the presence of DNA of *B. cockerelli* in the predator corpus. Individuals were randomly chosen from samples collected in the field in 2021 and 2022. The number of specimens of each species that was tested is shown (n).

After the PCR method was established to detect *B. cockerelli* DNA using lab-reared predators, we further applied it onto predators collected from fields. Out of the predators collected from the field, a total of 49 individuals from seven insect taxa were tested for the presence of *B. cockerelli* DNA. The results showed that *B. cockerelli* DNA was detected in 45% (n = 22) of the samples (Figure 3). Among the tested species, the Coleopteran predators exhibited the highest frequency of positive results for *B. cockerelli* DNA, with a rate of 60%. Specifically, *H. variegata* had the highest detection rate of 70%, followed by *C. transversalis*, with a detection rate of 62.5%. The Hemipteran predators *Nabis* sp. and *O. schellenbergii* (adults; unknown gender) tested positive on 38 and 17% of occasions, respectively. For Neuroptera, 30% of *M. signatus* larvae, the most abundant predator in the field survey, tested positive for *B. cockerelli* DNA. However, no significant difference was observed between positive and negative detections in the χ^2^ goodness-of-fit test.

## 4. Discussion

This study focused on sampling generalist predators in fields of Solanaceous crops in various locations in Western Australia, Australia. The findings revealed the presence of a diverse population of insect predator species, as well as spiders, within the agricultural ecosystem of Western Australia. These generalist predators may have potential to contribute to the control of the invasive pest *B. cockerelli*. Laboratory feeding trials and molecular analysis confirmed that a number of predators were utilizing *B. cockerelli* as a food source in the field.

The field samples included insects from Neuroptera, Coleoptera, Diptera and Hemiptera. Butler and Trumble [12] conducted a two-year field survey in southern California and identified Coleopteran and Hemipteran insects as key predators. In another study, Al-Jabr [54] identified two green lacewing species, *Chysoperla carnea* Stephens (Neuroptera: Chrysopidae) and *Chrysoperla rufilabris* Burmeister (Neuroptera: Chrysopidae), as having potential in the biological control of *B. cockerelli* in greenhouse tomato. The brown lacewing *Micromus tasmaniae* Walker (Neuroptera: Hemerobiidae) that we captured in 2022 has also been found in potato fields in New Zealand and can prey upon *B. cockerelli* [55,56]. Hemipteran and Dipteran predators were also sampled in our study. The predatory bug *Oechalia schellenbergii* Guérin (Heteroptera: Pentatomidae) was recorded in 2021 and 2022. While various Hemipteran predators have been reported to feed on *B. cockerelli* [12,57], no Diptera have yet been reported. Thus, there is a good number of potential predators that could contribute to the control of *B. cockerelli* in the Western Australian production systems.

The green lacewing (Neuroptera) was the most abundant predator in both sampling seasons, followed by Coccinellids. The highest number of predators was observed in a capsicum field in both 2021 and 2022. Available floral resources in capsicum field may support predators, especially adult lacewing survival, as Robinson et al. [58] observed in the case of the brown lacewing *M. tasmaniae* and buckwheat flowers in New Zealand. However, the presence of floral resources can affect omnivore predator–prey dynamics [59,60].

Despite the abundance of adult green lacewings and ladybirds, as well as of their eggs in the field, only a small number of lacewing larvae were collected, and no ladybird larvae were found. This may be due to the lacewing eggs being indirectly protected from cannibalism and intraguild predation, as they are laid at the tip of a thin hyaline stalk [61]. On the other hand, the predators at the larval stage may have experienced heavy intraguild predation, as they are not as mobile as the adults [58].

In our previous laboratory and glasshouse studies, the two most abundant ladybirds, *C. transversalis* and *H. variegata* [52,53], and the green lacewing *M. signatus* (our unpublished data) were confirmed as having potential for *B. cockerelli* control. Furthermore, during our field survey, *C. transversalis* larvae were observed preying on *B. cockerelli* nymphs [34]. This suggests that various other generalist predators might also contribute to the natural regulation of *B. cockerelli* populations, especially when the size of *B. cockerelli* populations surpasses that of other pest populations through resource competition. Therefore, field evaluation of these predators is required, as predation on the target pest may be affected by many factors, for example, alternative prey, intraguild predation, the surrounding vegetation through the provision of appropriate microclimates and overwintering sites for shelter, alternative food sources [62,63,64], etc.

Many different species of spider were sampled in both years of the field survey. Of these, Oxyopidae, a well-known predatory spider family [65,66], was the most common. The total number of spiders collected in 2022 was lower than in 2021, despite the larger number of sampling sites. This may be due to the smaller population of *B. cockerelli* in the fields in 2022 than in 2021. A similar relationship between the number of spiders and the number of *B. cockerelli* per plant was observed in bell pepper and tomato fields in southern California [12]. Although predatory spiders are not commercially available for augmentative release in crop fields, evidence for their occurrence in agroecosystems and their role in pest suppression is accumulating [67].

This is the first study to report the use of molecular techniques to identify predators of *B. cockerelli* in the field. The molecular method was developed to screen the sampled predator community to identify those predators that were consuming *B. cockerelli*. This could then guide the choice of species for future examination, for their development as biological control agents. In our time-since-feeding study, we observed that *B. cockerelli* DNA detection was not possible after 2 h for ladybirds and after 6 h for lacewing. This could be because lacewings took longer to digest *B. cockerelli* DNA than ladybirds, and thus the psyllid DNA remained detectable for longer. The difference in the time that DNA is detectable may need to be taken into account if a quantitative comparison of predation by different species is needed. Other possible limitations in the molecular analysis of field-collected corpora for prey DNA include the predator’s individual body size and the number of preys that it consumed [68,69], the risk of contamination [70] and temperature [71].

Overall, 45% of the field-collected generalist predators that were analysed contained *B. cockerelli* DNA in their corpus. The result shows that Coleopteran predators were positive most frequently (56% on average) than the other predators analysed, followed by Neuropta and the Hemiptera, although there were few samples for some species (2 to 10 samples). Coccinellid adults are well-known biocontrol agents, and many species are often used to suppress pest populations [72], including *B. cockerelli*, as reported in our previous studies [52,53]. Fewer *M. signatus* larvae tested positive for *B. cockerelli* DNA compared to adult ladybirds.

This may be due to the possibility that DNA may had already been digested due to a significant time elapsed between predation and predator sampling [32,37]. Alternatively, there might have been minimal to no consumption of the target prey on the sampling date. Despite the lower proportion of *M. signatus* that tested positive for psyllid DNA, the much greater number found in the field suggests that their impact on *B. cockerelli* populations is likely to be greater than that of ladybirds.

In conclusion, *M. signatus* and the Coccinellids’, particularly, *H. variegata* and *C. transversalis*, are promising options to further study as biological control agents for *B. cockerelli* in Western Australia. In addition, the PCR primers were highly reliable for detecting *B. cockerelli* DNA in a range of predator species and life stages and appear as a useful tool for identifying predators or monitoring their predation in the field. While biological control can contribute to manage *B. cockerelli* populations, the early detection of psyllid-vectored pathogens remains a crucial component of an integrated management strategy to minimize economic losses. Our results may contribute to the development of effective augmentative release options or inform conservation biological control strategies, both of which may contribute to integrated pest management of *B. cockerelli* in Australia.

## Figures and Tables

**Table 1:** Potential predators of *B. cockerelli* found in the fields of solanaceous crops sampled in 2021 and 2022.

Order	Family	Genus/Species	2021				2022									
			Site-1			Total	Site-1				Site-2	Site-3		Site-4	Site-5	Total
			Capsicum				Capsicum				Potato	Potato		Potato	Potato	
			1st Date (15.3)	2nd Date (22.3)	3rd Date (29.3)		1st Date (25.3)	2nd Date (1.4)	3rd Date (8.4)	4th Date (15.4)	1st Date (23.3)	1st Date (13.4)	2nd Date (11.11)	1st Date (11.11)	1st Date (11.11)	
Araneae	Araneidae	*Argiope protensa*	2	3		5		1					1			2
		*Backobourkia*			1	1										
	Oxyopidae	*Oxyopes*	3	24	1	28	2	4	1	9		3	2		1	22
	Salticidae			1		1	1			3						4
	Tetragnathidae	*Tetragnatha*		1		1		1								1
	Theridiidae	*Euryopes*		3		3										
	Thomisidae	*Thomisus spectabilis*		1		1		1								1
	Unidentified spider species		2	6	2	10	2	5		3						10
Diptera	Syrphidae	*Melangyna viridiceps*		1	1	2				1			2	2		5
Coleoptera	Coccinellidae	*Harmonia conformis*		7		7	1	2	1	1						5
		*Cheilomenes sexmaculata*		2		2		3		1						4
		*Coccinella transversalis*	2	41		43		2	2	1		1		1	1	8
		*Hippodamia variegata*	1	9		10		5	2	8		1	2	1	3	22
Hemiptera	Pentatomidae	*Oechalia schellenbergii*	2	7		9						2				2
	Miridae	*Creontiades* sp.	24	52		76						1				1
	Nabidae	*Nabis* sp.		31		31										
	Rhyparochromidae *Rhyparochromidae* sp.			3		3										
Neuroptera	Chrysopidae	*Mallada signatus*	Adult: 36	Adult: 356	Adult: 43	469	Adult: 38	Adult: 268	Adult: 68	Adult: 300			Adult: 7			859
			Larvae: 4	Larvae: 17	Larvae: 13		Larvae: 32	Larvae: 73	Larvae: 23	Larvae: 46			Larvae: 4			
		*Micromus tasmaniae*								Adult:1	Adult: 2	Adult: 1		Adult: 1		5

**Table 2:** The ability of the PCR test to detect *B. cockerelli* DNA in the predator’s corpus as a function of time since feeding *B. cockerelli*.

Predator Species	Time Since Feeding on *B. cockerelli*						
	0 h	0.5 h	1 h	2 h	6 h	12 h	24 h
*Coccinella transversalis* (adult)	**+**	**+**	**+**	**+**	**-**	**-**	**-**
*Hippodamia variegata* (adult)	**+**	**+**	**+**	**-**	**-**	**-**	**-**
*Mallada signatus* (larvae)	**+**	**+**	**+**	**+**	**+**	**-**	**-**

Note: “+”, positive detection of psyllid DNA; “-”, no detection of DNA.

## Data Availability

The datasets of the study will be made available upon request to the corresponding author.

## References

[1] Rosson P., Niemeyer M., Palma M., Ribera L. (2006). Economic Impacts of Zebra Chips on the Texas Potato Industry.

[2] Yang X.B., Liu T.X. (2009). Life history and life tables of *Bactericera cockerelli* (Homoptera: Psyllidae) on eggplant and bell pepper. Environ. Entomol..

[3] Munyaneza J.E. (2012). Zebra chip disease of potato: Biology, epidemiology, and management. Am. J. Potato Res..

[4] Mustafa T., Alvarez J.M., Munyaneza J.E. (2015). Effect of cyantraniliprole on probing behavior of potato psyllid, *Bactericera cockerelli* (Hemiptera: Triozidae), as measured by the electrical penetration graph technique. J. Econ. Entomol..

[5] Šulc K. (1909). *Trioza cockerelli* n. sp., a novelty from North America, being also of economic importance. Acta Soc. Entomol. Bohem..

[6] Department for Primary Industries and Regional Development (DPIRD) Tomato Potato Psyllid. https://www.agric.wa.gov.au/tomato-potato-psyllid-tpp.

[7] Munyaneza J.E., Crosslin J.M., Upton J.E. (2007). Association of *Bactericera cockerelli* (Homoptera: Psyllidae) with “zebra chip,” a new potato disease in southwestern United States and Mexico. J. Econ. Entomol..

[8] Goolsby J.A., Adamczyk J., Bextine B., Lin D., Munyaneza J.E., Bester G. (2007). Development of an IPM program for management of the potato psyllid to reduce incidence of zebra chip disorder in potatoes. Subtrop. Plant Sci..

[9] Olaniyan O., Rodríguez-Gasol N., Cayla N., Michaud E., Wratten S.D. (2020). *Bactericera cockerelli* (Šulc), a potential threat to China’s potato industry. J. Integr. Agric..

[10] Butler C.D., Trumble J.T. (2012). The potato psyllid, *Bactericera cockerelli* (Šulc) (Hemiptera: Triozidae): Life history, relationship to plant diseases, and management strategies. Terr. Arthropod Rev..

[11] EPPO (2013). *Bactericera* *cockerelli*. EPPO Bull..

[12] Butler C.D., Trumble J.T. (2012). Identification and Impact of Natural Enemies of *Bactericera cockerelli* (Hemiptera: Triozidae) in Southern California. J. Econ. Entomol..

[13] Guenthner J., Goolsby J., Greenway G. (2012). Use and cost of insecticides to control potato psyllids and zebra chip on potatoes. Southwest. Entomol..

[14] Jorgensen N., Butler R.C., Vereijssen J. (2013). Biorational insecticides for control of the tomato potato psyllid. N. Z. Plant Prot..

[15] Dich J., Zahm S.H., Hanberg A., Adami H.O. (1997). Pesticides and cancer. Cancer Causes Control.

[16] Geiger F., Bengtsson J., Berendse F., Weisser W.W., Emmerson M., Morales M.B., Ceryngier P., Liira J., Tscharntke T., Winqvist C. (2010). Persistent negative effects of pesticides on biodiversity and biological control potential on European farmland. Basic Appl. Ecol..

[17] Liu D., Trumble J.T. (2007). Comparative fitness of invasive and native populations of the potato psyllid (*Bactericera cockerelli*). Entomol. Exp. Appl..

[18] Vega-Gutierrez M.T., Rodríguez-Maciel J.C., Diaz-Gomez O., Bujanos-Muniz R., Mota-Sanchez D., Martínez-Carrillo J.L., Lagunes-Tejeda A., Garzon-Tiznado J.A. (2008). Susceptibility to insecticides in two Mexican populations of tomato-potato psyllid, *Bactericera cockerelli* (Šulc) (Hemiptera: Triozidae). Agrociencia.

[19] Prager S.M., Vindiola B., Kund G.S., Byrne F.J., Trumble J.T. (2013). Considerations for the use of neonicotinoid pesticides in management of *Bactericera cockerelli* (Šulk) (Hemiptera: Triozidae). Crop Prot..

[20] Cerna E., Ochoa Y., Aguirre L.A., Flores M., Landeros J. (2013). Determination of insecticide resistance in four populations of potato psyllid *Bactericera cockerelli* (Šulc) (Hemiptera: Triozidae). Phyton.

[21] Szczepaniec A., Varela K.A., Kiani M., Paetzold L., Rush C.M. (2019). Incidence of resistance to neonicotinoid insecticides in *Bactericera cockerelli* across Southwest U.S. Crop Prot..

[22] Hawkins B.A., Cornell H.V., Hochberg M.E. (1997). Predators, parasitoids, and pathogens as mortality agents in phytophagous insect populations. Ecology.

[23] Messelink G.J., Sabelis M.W., Janssen A. (2012). Generalist predators, food web complexities and biological pest control in greenhouse crops. Integrated Pest Management and Pest Control-Current and Future Tactics.

[24] Veronesi E.R., Olaniyan O., London H., Saville D.J., Wratten S.D. (2021). Potential inter-guild interactions to enhance biological control of *Bactericera cockerelli* on tomatoes: A laboratory and cage study. BioControl.

[25] Shea K., Chesson P. (2002). Community ecology theory as a framework for biological invasions. Trends Ecol. Evol..

[26] Bulgarini G., Piemontese L., Guidetti R., Cesari M., di Bella E., Maistrello L. (2021). Identification of Predatory Arthropods of the Invasive *Halyomorpha halys* through Molecular Gut Content Analysis. Agric. For. Entomol..

[27] Harwood J.D., Desneux N., Yoo H.J., Rowley D.L., Greenstone M.H., Obrycki J.J., O’Neil R.J. (2007). Tracking the role of alternative prey in soybean aphid predation by *Orius insidiosus*: A molecular approach. Mol. Ecol..

[28] Desneux N., O’Neil R.J. (2008). Potential of an alternative prey to disrupt predation of the generalist predator, *Orius insidiosus*, on the pest aphid, *Aphis glycines*, via short-term indirect interactions. Bull. Entomol. Res..

[29] Juen A., Hogendoorn K., Ma G., Schmidt O., Keller M.A. (2012). Analysing the diets of invertebrate predators using terminal restriction fragments. J. Pest Sci..

[30] Chen X., Stansly P.A. (2014). Biology of *Tamarixia radiata* (Hymenoptera: Eulophidae), Parasitoid of the Citrus Greening Disease Vector *Diaphorina citri* (Hemiptera: Psylloidea): A Mini Review. Fla. Entomol..

[31] Tanaka S., Nishida T., Ohsaki N. (2007). Sequential rapid adaptation of indigenous parasitoid wasps to the invasive butterfly *Pieris brassicae*. Evolution.

[32] Symondson W.O., Sunderland K.D., Greenstone M.H. (2002). Can generalist predators be effective biocontrol agents?. Annu. Rev. Entomol..

[33] Eubanks M.D., Denno R.F. (2000). Health food versus fast food: The effects of prey quality and mobility on prey selection by a generalist predator and indirect interactions among prey species. Ecol. Entomol..

[34] Sarkar S.C., Hatt S., Philips A., Akter M., Milroy S.P., Xu W. (2023). Tomato Potato Psyllid *Bactericera cockerelli* (Hemiptera: Triozidae) in Australia: Incursion, Potential Impact and Opportunities for Biological Control. Insects.

[35] Symondson W.O.C. (2002). Molecular identification of prey in predator diets. Mol. Ecol..

[36] Greenstone M.H., Szendrei Z., Payton M.E., Rowley D.L., Coudron T.C., Weber D.C. (2010). Choosing natural enemies for conservation biological control: Use of the prey detectability half-life to rank key predators of Colorado potato beetle. Entomol. Exp. Appl..

[37] Dhami M.K., Dsouza M., Waite D.W., Anderson D., Li D. (2016). Real-Time PCR Assay for the Identification of the Brown Marmorated Stink Bug (*Halyomorpha halys*). Front. Mol. Biosci..

[38] Unruh T.R., Miliczky E.R., Horton D.R., Thomsen-Archer K., Rehfield-Ray L., Jones V.P. (2016). Gut content analysis of arthropod predators of codling moth in Washington apple orchards. Biol. Control.

[39] Casey J.M., Meyer C.P., Morat F., Brandl S.J., Planes S., Parravicini V. (2019). Reconstructing Hyperdiverse Food Webs: Gut Content Metabarcoding as a Tool to Disentangle Trophic Interactions on Coral Reefs. Methods Ecol. Evol..

[40] Siegenthaler A., Wangensteen O.S., Soto A.Z., Benvenuto C., Corrigan L., Mariani S. (2019). Metabarcoding of shrimp stomach content: Harnessing a natural sampler for fish biodiversity monitoring. Mol. Ecol. Resour..

[41] Bouvet J.P., Urbaneja A., Pérez-Hedo M., Monzó C. (2019). Contribution of Predation to the Biological Control of a Key Herbivorous Pest in Citrus Agroecosystems. J. Anim. Ecol..

[42] Jacobsen S.K., Sigsgaard L., Hansen K., Harwood J.D., Chapman E.G., Hurtado M.A., Jensen A.B. (2019). Generalist predator contributions to the control of *Tetranychus urticae* in strawberry crops documented by PCR-based gut content analysis. Exp. Appl. Acarol..

[43] Hoogendoorn M., Heimpel G.E. (2001). PCR-Based Gut Content Analysis of Insect Predators: Using Ribosomal ITS-1 Fragments from Prey to Estimate Predation Frequency. Mol. Ecol..

[44] Harwood J.D., Yoo H.J., Greenstone M.H., Rowley D.L., O’Neil R.J. (2009). Differential impact of adults and nymphs of a generalist predator on an exotic invasive pest demonstrated by molecular gut-content analysis. Biol. Invasions.

[45] Roubinet E., Jonsson T., Malsher G., Staudacher K., Traugott M., Ekbom B., Jonsson M. (2018). High redundancy as well as complementary prey choice characterize generalist predator food webs in agroecosystems. Sci. Rep..

[46] Rondoni G., Athey K.J., Harwood J.D., Conti E., Ricci C., Obrycki J.J. (2015). Development and application of molecular gut-content analysis to detect aphid and coccinellid predation by *Harmonia axyridis* (Coleoptera: Coccinellidae) in Italy. Insect Sci..

[47] Harper G.L., King R.A., Dodd C.S., Harwood J.D., Glen D.M., Bruford M.W., Symondson W.O. (2005). Rapid screening of invertebrate predators for multiple prey DNA targets. Mol. Ecol..

[48] Zhang G.F., Lü Z.C., Wan F.H. (2007). Detection of *Bemisia tabaci* remains in predator guts using a sequence-characterized amplified region marker. Entomol. Exp. Appl..

[49] Peterson J.A., Burkness E.C., Harwood J.D., Hutchison W.D. (2018). Molecular gut-content analysis reveals high frequency of *Helicoverpa zea* (Lepidoptera: Noctuidae) consumption by *Orius insidiosus* (Hemiptera: Anthocoridae) in sweet corn. BioControl.

[50] Chapman R.I., Strube L., Bextine B. (2010). Population Genetics of the Potato Psyllid: Impacts on Zebra Chip Epidemiology. Proceedings of the 10th Annual Zebra Chip Reporting Session.

[51] Wu F., Cen Y., Wallis C.M., Trumble J.T., Prager S., Yokomi R., Zheng Z., Deng X., Chen J., Liang G. (2016). The complete mitochondrial genome sequence of *Bactericera cockerelli* and comparison with three other Psylloidea species. PLoS ONE.

[52] Sarkar S.C., Milroy S.P., Xu W. (2022). Development and reproduction of a native generalist predator, *Coccinella transversalis* (Coleoptera: Coccinellidae), on the tomato potato psyllid, *Bactericera cockerelli*, with a greenhouse assay of biocontrol potential. Biol. Control.

[53] Sarkar S.C., Milroy S.P., Xu W. (2023). Potential of variegated lady beetle *Hippodamia variegata* in management of invasive tomato potato psyllid *Bactericera cockerelli*. Pest Manag. Sci..

[54] Al-Jabr A.M. (1999). Integrated Pest Management of Tomato Potato Psyllid, *Paratrioza cockerelli* (Sulc) (Homoptera: Psyllidae) with Emphasis on Its Importance in Greenhouse Grown Tomatoes. Ph.D. Dissertation.

[55] Walker G.P., MacDonald F.H., Larsen N.J., Wallace A.R. (2011). Monitoring *Bactericera cockerelli* and associated insect populations in potatoes in South Auckland. N. Z. Plant Prot..

[56] MacDonald F.H., Connolly P.G., Larsen N.J., Walker G.P. (2016). The voracity of five insect predators on *Bactericera cockerelli* (Sülc) (Hemiptera: Triozidae) (tomato potato psyllid; TPP). N. Z. Entomol..

[57] Knowlton G.F., Allen M. (1936). Three hemipterous predators of the potato psyllid. Proc. Utah Acad. Sci..

[58] Robinson K.A., Jonsson M., Wratten S.D., Wade M.R., Buckley H.L. (2008). Implications of floral resources for predation by an omnivorous lacewing. Basic Appl. Ecol..

[59] Gurr G., Wratten S., Tylianakis J., Kean J., Keller M. (2005). Providing plant foods for natural enemies in farming systems: Balancing practicalities and theory. CABI Agric. Biosci..

[60] Van Rijn P.C., Sabelis M.W., Wäckers F.L., van Rijn P.C.J., Bruin J. (2005). Impact of plant-provided food on herbivore-carnivore dynamics. Plant-Provided Food and Herbivore–Carnivore Interactions.

[61] Hayashi M., Nomura M. (2014). Eggs of *Mallada desjardinsi* (*Neuroptera*: Chrysopidae) are protected by ants: The role of egg stalks in ant-tended aphid colonies. Environ. Entomol..

[62] Gurr G.M., Wratten S.D., Landis D.A., You M. (2017). Habitat management to suppress pest populations: Progress and prospects. Annu. Rev. Entomol..

[63] Shields M.W., Johnson A.C., Pandey S., Cullen R., González-Chang M., Wratten S.D., Gurr G.M. (2019). History, current situation and challenges for conservation biological control. Biol. Control.

[64] Gardarin A., Pigot J., Valantin-Morison M. (2021). The hump-shaped effect of plant functional diversity on the biological control of a multi-species pest community. Sci. Rep..

[65] Young O.P., Lockley T.C. (1985). The striped lynx spider, *Oxyopes salticus* (Araneae: Oxyopidae), in agroecosystems. Entomophaga.

[66] Nyffeler M., Dean D.A., Sterling W.L. (1987). Evaluation of the importance of the striped lynx spider, *Oxyopes salticus* (Araneae: Oxyopidae), as a predator in Texas cotton. Environ. Entomol..

[67] Michalko R., Pekár S., Entling M.H. (2019). An updated perspective on spiders as generalist predators in biological control. Oecologia.

[68] Eitzinger B., Unger E.M., Traugott M., Scheu S. (2014). Effects of prey quality and predator body size on prey DNA detection success in a centipede predator. Mol. Ecol..

[69] Eitzinger B., Rall B.C., Traugott M., Scheu S. (2018). Testing the validity of functional response models using molecular gut content analysis for prey choice in soil predators. Oikos.

[70] Symondson W.O.C. (2012). The molecular revolution: Using polymerase chain reaction-based methods to explore the role of predators in terrestrial food webs. Biodivers. Insect Pests Key Issues Sustain. Manag..

[71] Greenstone M.H., Payton M.E., Weber D.C., Simmons A.M. (2014). The detectability half-life in arthropod predator-prey research: What it is, why we need it, how to measure it, and how to use it. Mol. Ecol..

[72] Rutledge C.E., O’Neil R.J., Fox T.B., Landis D.A. (2004). Soybean aphid predators and their use in Integrated Pest Management. Ann. Entomol. Soc. Am..

